# Saliva Microbiome Changes in Patients With Periodontitis With and Without Chronic Obstructive Pulmonary Disease

**DOI:** 10.3389/fcimb.2020.00124

**Published:** 2020-04-15

**Authors:** Mei Lin, Xuefen Li, Jitian Wang, Cheng Cheng, Tianyi Zhang, Xiaozhe Han, Yiqing Song, Zuomin Wang, Songlin Wang

**Affiliations:** ^1^Department of Stomatology, Beijing Chao-Yang Hospital Affiliated to Capital Medical University, Beijing, China; ^2^Salivary Gland Disease Center and Beijing Key Laboratory of Tooth Regeneration and Function Reconstruction, School of Stomatology, Capital Medical University, Beijing, China; ^3^Central Laboratory, Peking University School and Hospital of Stomatology, Beijing, China; ^4^School of Stomatology, Shanxi Medical University, Taiyuan, China; ^5^Department of Immunology and Infectious Disease, The Forsyth Institute, Cambridge, MA, United States; ^6^Department of Epidemiology, Fairbanks School of Public Health, Indiana University, Indianapolis, IN, United States; ^7^Department of Biochemistry and Molecular Biology, School of Basic Medicine, Capital Medical University, Beijing, China

**Keywords:** saliva, microbiota, bacterial, periodontitis, COPD

## Abstract

**Objective:** The oral microbiota plays a key part in the initial colonization by pathogens and the chronic inflammatory reaction of the host. We measured variations in the salivary microbiota and evaluated their potential associations with periodontitis and chronic obstructive pulmonary disease (COPD).

**Methods:** We investigated the salivary microbiota of patients with COPD and periodontitis (*n* = 21) compared with that in patients with periodontitis alone (*n* = 36) and with healthy controls (HCs; *n* = 14), using pyrosequencing of polymerase chain reaction-amplified 16s rRNA genes.

**Results:** Bacterial richness and diversity were significantly higher in patients suffering from COPD, and the bacterial family Lachnospiraceae was observed frequently only among patients with COPD and periodontitis. *Veillonella, Rothia, Actinomyces*, and *Fusobacterium* were the core bacterial genera that showed significant differences among patients with coincident COPD and periodontitis, patients with periodontitis alone, and HCs (*p* < 0.05). *Veillonella, Rothia*, and *Actinomyces* were observed much more frequently in patients with COPD and periodontitis, compared with that in HCs. All tested populations were divided into subgroups based on sex, smoking, or periodontitis index. In the subgroup with a bleeding index >2, *Rothia* was significantly different in periodontitis with and without COPD groups compared with HCs. In the subgroup with a plaque index >2.5, *Rothia* and *Veillonella* showed significant differences in periodontitis with and without COPD groups compared with HCs.

**Conclusion:** Variations in salivary microbiota may be associated with COPD and periodontitis.

## Introduction

Chronic obstructive pulmonary disease (COPD) is characterized by progressive airflow obstruction produced by a chronically increased inflammatory response within the airways. COPD is associated with high morbidity and mortality (Sapey et al., 2008).

Recent research has revealed the importance of extra-pulmonary effects on treatment, and the contribution of comorbidities, to the overall severity of COPD (Vogelmeier et al., 2017). Much of the financial cost associated with COPD treatment is related to common systemic comorbidities, which are thought to be part of organ-specific inflammatory processes, for example, respiratory inflammation, cardiovascular disease and, most commonly, periodontitis (Du et al., 2016). Microorganisms are thought to be one of the main etiologic factors that exacerbate COPD. Identification of the potential risk factors that contribute to COPD pathogenesis may reveal interventions that prevent and/or delay initiation or slow COPD progression. Bacteria in healthy lung tissue originate from the air, direct mucosal dispersion, and aspiration (Dickson et al., 2014) but the exacerbating pathogens of COPD are not known.

Periodontitis is the chronic inflammation of the gums and supporting structures of the teeth. It results in failure of function of the supporting connective tissue and the bone of teeth. Oral pathogens from periodontal lesions and inflammatory cytokines induce systemic inflammation that may contribute to COPD pathogenesis (Terpenning, 2001). Salivary bacteria are an integral part of the oral microbiota and have been characterized in healthy individuals (Segata et al., 2012). More than 700 bacterial species have been recognized in the oral cavity (Dewhirst et al., 2010). Alterations in the salivary microbiota related to periodontitis and dental caries are far more complex than previously believed, as assessed using microarray-based methods (Preza et al., 2009) and sequencing (Wu et al., 2017). COPD is associated with periodontitis *via* significant changes in specific bacteria (Obata et al., 2014).

We compared the microbiota in human saliva from healthy controls (HCs) and patients with periodontitis with and without COPD using 16s rRNA gene sequencing and evaluated the potential associations between periodontitis and COPD. The establishment and potential translational utility of “salivary microbial signatures” could be used to identify additional biomarkers for the non-invasive detection of COPD.

## Materials and Methods

### Patients

Seventy-one participants were recruited between 2015 and 2016 from the Beijing Chao-Yang Hospital. According to a clinical assessment of their periodontal health and lung function, study participants were divided into three groups: (i) HCs; (ii) patients with periodontitis; (iii) patients with periodontitis and COPD. Participants with a history of other inflammatory diseases were excluded.

### Periodontal Examination

Two trained dentists carried out periodontal examinations. Examinations were repeated throughout the study to assess intra-examiner reliability. The evaluation comprised periodontal probing, oral hygiene, examination for dental caries, and mucosal evaluation. Clinical measurements of periodontal parameters were recorded on a full-mouth basis: probing depth (PD), plaque index (PLI), bleeding index (BI), attachment loss (AL), abscess, and teeth movement. All assessments were undertaken at two sites per tooth.

According to the survey methods for basic oral health designed by the World Health Organization, sites were air-dried before measurement. Examiners used Williams probes with an intensity of ≤25 g to determine the periodontitis classification. “Mild” periodontitis was at least one tooth with a PD ≥3 mm and AL ≥3 mm, or PD ≥4 mm and AL ≥3 mm ≤30%. “Moderate” periodontitis was a PD ≥5 mm and AL ≥4-mm teeth <30%, or teeth with a PD ≥4 mm and AL ≥3 mm between 30 and 60%. “Severe” periodontitis was a PD ≥5 mm and AL ≥4-mm teeth ≥30%; or a PD ≥4 mm and AL ≥3-mm teeth ≥60%. Only patients with moderate periodontitis and severe periodontitis were selected in the present study.

### Diagnosis of COPD

Criteria for the diagnosis of COPD were based on the spirometry guidelines set by the Global Initiative for Chronic Obstructive Lung Disease (Global Strategy for the Diagnosis, Management, and Prevention of Chronic Obstructive Pulmonary Disease, Update 2009).

A trained technician evaluated lung function using spirometry. Pulmonary evaluation was based on the forced expiratory volume in 1 s/forced vital capacity (FEV_1_/FVC) × 100. The percent predicted FEV_1_ was used to categorize severity.

COPD was classified as: “mild” (normal pulmonary function); “moderate” [FEV_1_/FVC < 70%, FEV_1_ ≥ 80% of the expected value, with or without chronic symptoms (cough, sputum)]; “severe” (moderate FEV_1_/FVC < 70%, 30% ≥ FEV < 80% of the expected value); “very severe” (severe FEV_1_/FVC <70%, FEV_1_ < 30% of the expected value, or FEV_1_ < 50% of the expected value accompanied by clinical symptoms of pulmonary-function failure or right-ventricular failure).

### Sample Collection

Saliva samples were collected from each participant. According to a well-established protocol, saliva samples were collected between 8:00 and 11:00 AM and before any dental treatment was performed to avoid bleeding and contamination of saliva. Participants started by flushing thoroughly with water, and expectorated unstimulated 3 to 5 ml saliva into a 10 ml sterile Eppendorf tube which was immediately stored at −80°C. Each saliva sample was labeled with a unique identification number.

### DNA Extraction and Amplification by Polymerase Chain Reaction (PCR)

Genomic DNA was extracted from human saliva samples using a Ready-Lyse™ Lysozyme Solution (R1802M; Epicentre, San Diego, CA, USA) and a MasterPure DNA Purification kit (MCD85201; Epicentre) according to manufacturer's instructions. The quality of the extracted DNA was checked using 1% agarose-gel electrophoresis and spectrophotometry (ratio of absorbance at 260/280 nm). Samples of extracted DNA were stored at −20°C for further analyses.

The V3–V4 hypervariable regions of the bacterial 16S rRNA gene were subjected to high-throughput sequencing by Beijing Allwegene Tech (Beijing, China) using the Miseq™ PE300 sequencing platform (Illumina, San Diego, CA, USA). The V3–V4 region of the bacterial 16S rRNA gene was amplified with the universal forward primer 338F (5′-ACTCCTACGGGAGGCAGCAG-3′) and reverse primer 806R (5′-GACTACHVGGGTWTCTAAT-3′). These primers contained a set of 8-nucleotide barcode sequences unique to each sample. The PCR program was: 95°C for 5 min; 25 cycles at 95°C for 30 s, 55°C for 30 s, and 72°C for 30 s; with a final extension of 72°C for 10 min. PCRs were carried out in triplicate using a 25-μL reaction mixture containing 2.5 μl of 10× Pyrobest™ Buffer, 2 μl of 2.5 mM dNTPs, 1 μl of each primer (10 μM), 0.4 U of Pyrobest DNA Polymerase (TaKaRa Biotechnology, Shiga, Japan), and 15 ng of template DNA. The amplicon mixture was applied to a MiSeq Genome Sequencer (Illumina).

### Sequencing

Amplicons were extracted from 2% agarose gels and purified using the AxyPrep™ DNA Gel Extraction kit (Axygen Biosciences, Union City, CA, USA) according to the manufacturer's instructions and quantified using QuantiFluor™-ST (Promega, Fitchburg, WI, USA). Purified amplicons were pooled in equimolar and paired-end sequences (2 × 300) on the MiSeq platform according to standard protocols.

### Processing of Sequencing Data

Extraction of high-quality sequences was done first using Quantitative Insights Into Microbial Ecology (QIIME) v1.2.1 (http://www.qiime.org/) (Caporaso et al., 2010). Raw sequences were selected based on the sequence length, quality, primer, and tag. Low-quality sequences were removed, including: (i) raw reads shorter than 110 nucleotides; (ii) 300-bp reads that were truncated at any site receiving an average quality score <20 over a 50-bp sliding window, and discarding truncated reads shorter than 50 bp; (iii) exact matching of barcodes, two-nucleotide mismatches in primer matching, and reads containing ambiguous characters were removed; (iv) only sequences that overlapped for >10 bp were assembled according to their overlap sequence. Reads that could not be assembled were discarded.

The unique sequence set was classified into operational taxonomic units (OTUs) under the threshold of 97% identity using QIIME UCLUST. Chimeric sequences were identified and removed using Usearch v8.0.1623. The taxonomy of each 16S-rRNA gene sequence was analyzed by UCLUST against the Silva119 16S rRNA database (Cole et al., 2009) using a confidence threshold of 90%.

### Statistical Analyses

Data analyses were undertaken using SPSS v19 (IBM, Armonk, NY, USA). Group comparisons for continuous variables with normal distributions were carried out using analysis of variance (ANOVA). Categorical variables were compared using the chi-squared test. *P* < 0.05 was considered significant (in two-sided tests). All populations were divided into subgroups based on sex, smoking, BI, and PLI.

**Figure 1:**
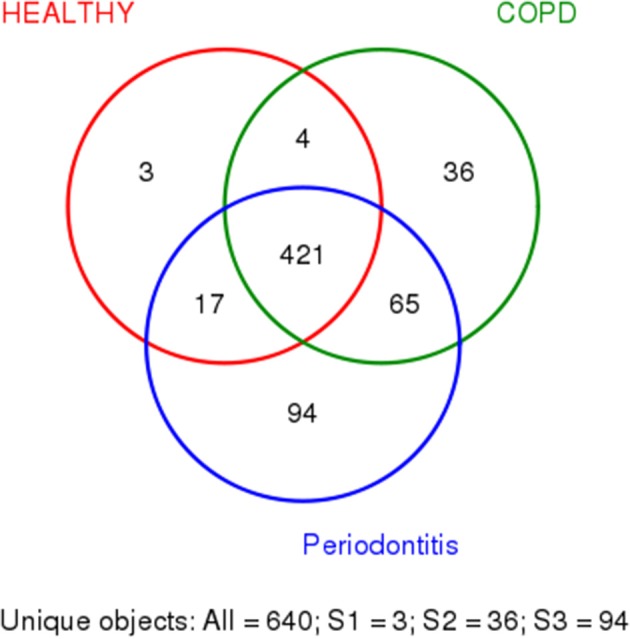
Shared and unique bacteria in patients with periodontitis (P), patients with periodontitis, and COPD (C+P), and healthy controls (H). The different colors represent different groups. The overlap represents shared bacteria. There are 640 unique objects, and 421 appear in all three groups.

We used R (v3.6.0) to draw Venn diagram to depict OTUs that were unique or shared to the three groups (Figure 1) (Fouts et al., 2012).

**Figure 2:**
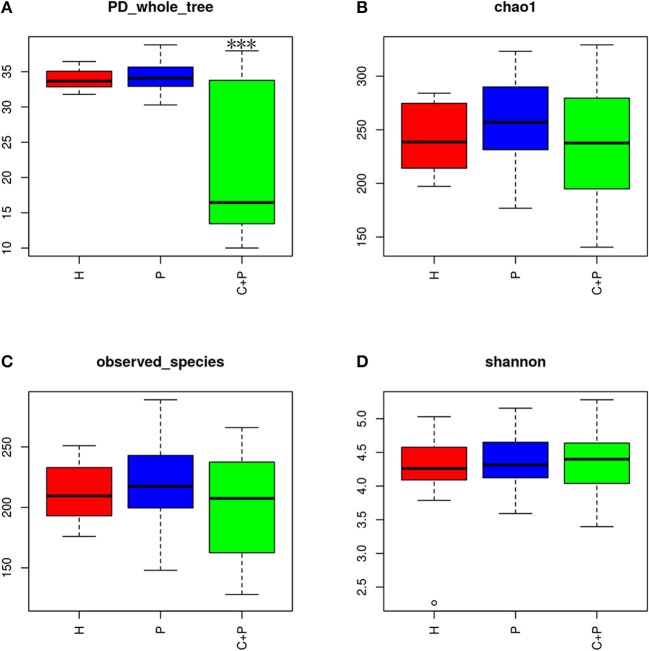
Alpha diversity of the saliva microbiome in patients with periodontitis (P), patients with periodontitis, and COPD (C+P), and healthy controls (H). **(A)** The phylogenetic diversity (PD) whole tree is a kind of diversity index calculated based on a phylogenetic tree. The *** shows significant differences (*P* < 0.05) tested using ANOVA in the PD whole tree index. **(B)** Chao1 is an abundance index and **(D)** Shannon is a diversity index. **(C)** Observed species is the index of all amount of OTUs detected.

Alpha diversity was calculated on the basis of the gene profile for each sample based on the Shannon, Chao1, Simpson, and PD_whole_tree's index. Alpha-diversity estimates were computed using QIIME1 (v1.8.0) software (Figure 2) (Schloss et al., 2011).

Non-metric multidimensional scaling (NMDS) is an indirect gradient analysis which produces an ordination based on a distance or dissimilarity matrix. Different from the method which is used to maximize the variance or correspondence between objects in an ordination, NMDS was used to represent, as closely as possible, the pairwise dissimilarity between objects in a low-dimensional space. Any dissimilarity coefficient or distance measurement may be used to build the distance matrix. We used the R (v3.6.0), vegan package for the calculation of Bray- curtis distance matrix and the ggplot2 package for NMDS analysis (**Figure 4**) (Noval Rivas et al., 2013).

**Figure 3:**
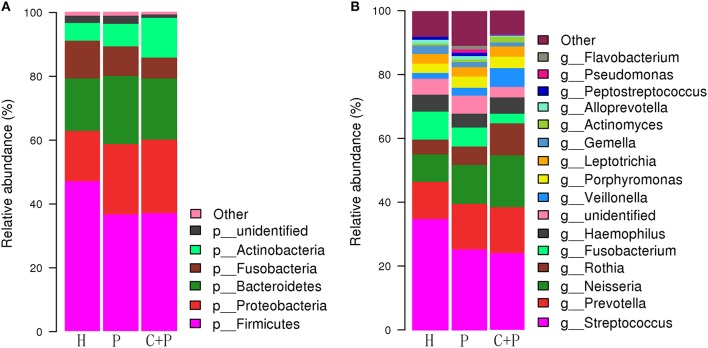
Relative abundance of major species in healthy controls (H), patients with periodontitis (P), and patients with both periodontitis and COPD (C+P). **(A,B)** shows the relative abundance of the bacterial species at the genus and phylum levels, respectively. Each column of the bar graph represents a group, and each patch represents a class of microorganisms in this group in proportion. <1% of the species were classified as other. OTUs were annotated by default QIIME UCLUST method with default settings and the GreenGene database.

Based on the relative abundance and Species annotation results, R (v3.6.0) software was used for the analysis of species composition histogram and changing trend histogram of specific bacteria among groups (Figures 3, **6**) (Oberauner et al., 2013).

LEfSe (Linear discriminant analysis Effect Size) determines the bacterial species most likely to explain differences between classes by coupling standard tests for statistical significance with additional tests encoding biological consistency and effect relevance. First, Kruskal-Wallis (KW) was used to detect features with significant differential abundance with respect to the class of interest with a significance set to 0.05; Second, biological consistency is subsequently investigated using a set of pairwise tests among subclasses, using the Wilcoxon rank-sum test, significance was set to 0.05; Finally, LDA was used to estimate the effect size of each differentially abundant feature and, if desired by the investigator, to perform dimension reduction. We set the LDA significance at 4 (**Figure 5**) (Segata et al., 2011).

Forest plots were drawn with R v3.6.1 (R, Vienna, Austria). Unless otherwise noted, all tests of differences between groups and other factors were conducted at a 2-sided alpha level of 0.05. Group comparisons for continuous variables with normal distributions were assessed by an analysis of variance (ANOVA). The group differences from an ANOVA model with groups were also calculated. Categorical variables were compared using the chi-squared test. Descriptive statistics were provided for patient demographics and all baseline characteristics. All continuous variables were summarized using descriptive statistics, including the number of observations (n), mean, and standard deviation (SD). All categorical variables were summarized using the number and percent of subjects. The primary analysis is the differences in the phylogenetic diversity of bacteria between groups. For further study, linear discriminant analysis (LDA) coupled with effect size (LEfSe) analyses with an LDA value of 3.0 were used to investigate the differences in microbial communities in the different groups. All populations were divided into subgroups based on sex, smoking, BI, and PLI to show the differences in the phylogenetic diversity of bacteria between groups. A forest plot clarifying the relationship between bacterial and clinical symptoms across subgroups was generated (**Figures 7**, **8**).

## Results

### Demographic Data

The 71 participants were divided into three groups according to the presence of periodontitis or COPD. There were no significant differences in sex, age, or body mass index (BMI) among the groups (Table 1). Among the three groups, the mean age of participants ranged from 59.75 to 60.36 years; and mean BMI ranged from 23.83 to 25.07 kg/m^2^.

**Table 1:** Basic characteristics of participants.

	**Group 1** **(normal controls)**	**Group 2** **(periodontitis)**	**Group 3** **(COPD and periodontitis)**	***P***
*N*	14	36	21	^a^
Gender				0.78^a^
Gender (male, %)	7 (50%)	16 (44.4%)	12 (57.1%)	
Age, years	60.36 ± 8.78	59.75 ± 8.00	60.24 ± 8.84	0.96^b^
BMI, kg/m^2^	24.80 ± 4.70	25.07 ± 2.65	23.83 ± 3.47	0.41^b^
Smoking status, %				0.005^a^
Current	0 (0%)	6 (16.67%)	3 (14.29%)	
Former	2 (14.29%)	6 (16.67%)	12 (57.14%)	
Never	12 (85.71%)	24 (66.67%)	6 (28.57%)	
Periodontal parameter
PD	2.25 ± 0.44	3.79 ± 0.76	3.56 ± 0.38	<0.001^b^
PLI	2.05 ± 0.78	2.63 ± 0.54	2.81 ± 0.35	0.001^b^
BI	1.68 ± 0.47	2.85 ± 0.54	2.62 ± 0.51	<0.001^b^
AL	1.14 ± 0.53	4.70 ± 1.64	5.42 ± 2.04	<0.001^b^
Lung function
FEV1%	93.32± 16.2	98.32± 14.93	42.13 ± 11.63	<0.001^b^
FEV1/FVC	77.91± 7.06	80.93± 5.81	47.15 ± 8.11	<0.001^b^

*P-values for the differences between the groups from ^a^chi-squared test and ^b^ANOVA. BMI, Body Mass Index; PD, probing depth; PLI, plaque index; BI, bleeding index; Al, alveolar bone lose; FEV1%, forced expiratory volume after 1 s; FEV1/FVC, forced expiratory volume after 1 s (FEV1)/forced vital capacity (FVC) × 100*.

Patients with periodontitis and COPD were more likely to be men. As expected, patients with COPD were more likely to be smokers (former and current smokers). Patients with periodontitis (with or without COPD) had a higher periodontal index (PD, BI, PLI, AL) compared with that in HCs, and there was no significant difference between patients with periodontitis and COPD, or patients with periodontitis alone (Table 1).

### Bacterial Composition in Saliva in Patients With Periodontitis With and Without COPD and in HCs

In Figure 1, the colors represent different groups, with the overlap representing shared bacteria. Among the 640 OTUs detected in the 71 samples, 421 were found in all three groups. Three OTUs were found only in HCs, 94 were found only in patients with periodontitis alone, and 36 were found only in patients with COPD and periodontitis. Four OTUs were found in HCs and patients with COPD and periodontitis, 17 were shared by HCs and patients with periodontitis only, and 65 were shared by patients with periodontitis and those with periodontitis and COPD (Figure 1). Among all the bacteria that appeared only in one group, Lachnospiraceae was observed most frequently in samples from the COPD + periodontitis group, suggesting that they newly colonize only people with COPD and periodontitis.

### Significant Differences in the Phylogenetic Diversity of Bacteria From Patients With COPD and Periodontitis Compared With That From Patients With Periodontitis and HCs

Alpha-diversity analyses showed significant differences in phylogenetic diversity (PD whole tree) for samples from patients with periodontitis and COPD (ANOVA, *p* < 0.05) (Figure 2A). A decrease in abundance (Chao1) and an increase in diversity (Shannon) were observed in patients with periodontitis and COPD compared with those in the other two groups, but neither of the differences was statistically significant (ANOVA, *p* > 0.05) (Figures 2B,D). A significant difference in the richness of bacterial species was not observed among three groups (ANOVA, *p* > 0.05) (Figure 2C).

### Salivary Microbiota Composition and Abundance in HCs and Patients With Periodontitis With and Without COPD

We found that the bacterial phyla Firmicutes (46.97%), Bacteroidetes (16.47%), Proteobacteria (15.7%), Fusobacteria (11.79%), and Actinobacteria (5.56%) constituted most of the healthy salivary microbiota. In patients with periodontitis only, they were ranked differently: Firmicutes (36.56%), Proteobacteria (22.01%), Bacteroidetes (21.37%), Fusobacteria (9.27%), and Actinobacteria *(*7.04%). In patients with periodontitis and COPD, the major bacterial phyla were Firmicutes (36.91%), Proteobacteria (23.06%), Bacteroidetes (19.23%), Actinobacteria (12.43%), and Fusobacteria (6.49%) (Figure 3).

In summary, Firmicutes was the most abundant phylum in all three groups. However, Proteobacteria was secondary only in the periodontitis group, and Actinobacteria was much more abundant in patients with periodontitis and COPD than in those with periodontitis only.

### Changes in the Composition and Diversity of Phylogenetic Species in the Salivary Microbiota of HCs and Patients With Periodontitis With and Without COPD

**Figure 4:**
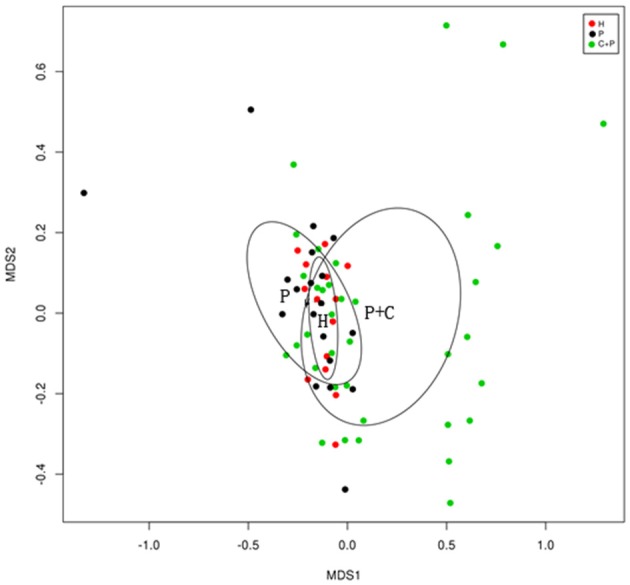
NMDS (Non-metric multidimensional scaling) showing bacterial differences among groups. NMDS analysis based on unweighted UniFrac distance matrices shows samples from healthy controls (H), patients with periodontitis (P), and patients with periodontitis and COPD (C+P) clustered together separately. The bacterial C+P group is separated from the others, and the healthy control group is shown by a smaller circle within the simple periodontitis circle.

Beta-diversity analyses showed distinct differences in the salivary microbiota of patients with periodontitis and COPD compared with the microbiota from patients with periodontitis only and HCs. In a non-metric multidimensional scaling (NMDS) analysis, the spatial distance indicates the extent of the difference. The periodontitis + COPD group was far from the other two groups (Figure 4), indicating that the salivary microbiota of patients with periodontitis and COPD was significantly different from that of patients with periodontitis only and HCs. The two circles for periodontitis alone and HCs overlapped each other on the map, which also indicated that the periodontitis-only group had a similar bacterial composition to that of HCs (though with greater diversity).

### Significant Differences in the Salivary Microbiota Between HCs and Patients With Periodontitis With and Without COPD

**Figure 5:**
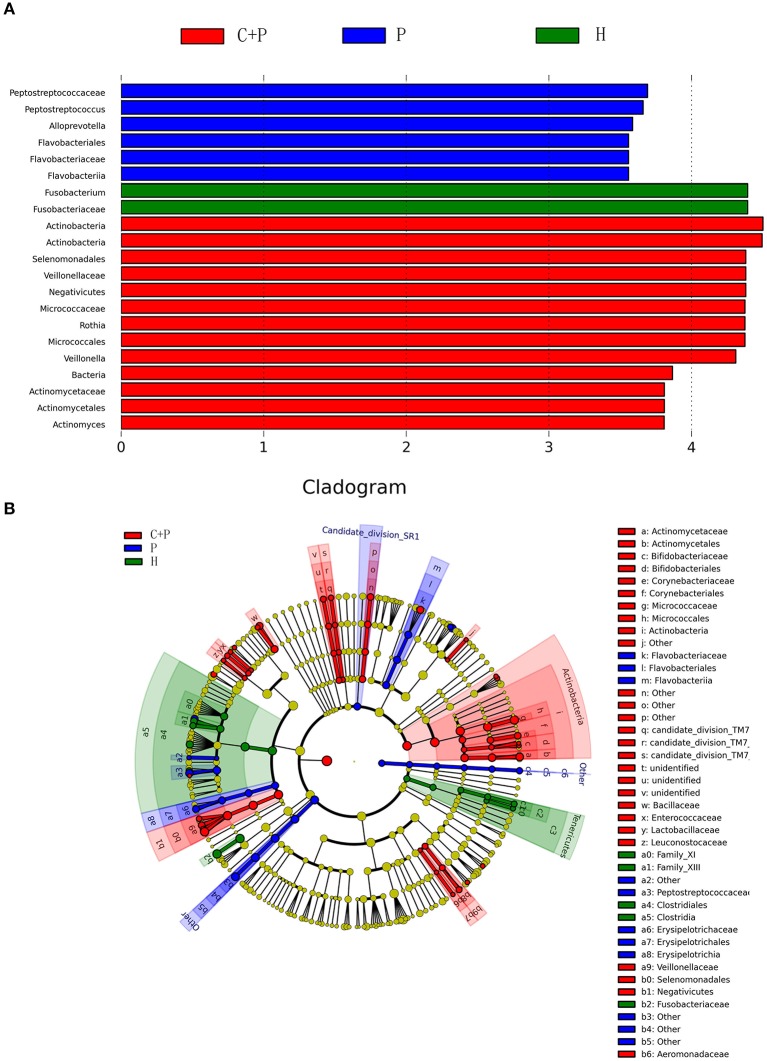
Partial bacterial taxa that differed significantly among healthy controls (H), patients with periodontitis (P), and patients with both periodontitis and COPD (C+P), according to linear discriminant analysis coupled with effect size (LEfSe). **(A)** Shows bacterial clades that are differentially abundant in group P (blue), group C+P (red), and group H (green). Clades in this graph were both statistically significant (*P* < 0.05) and had a linear discriminants (LDs) score > ±3, which is considered a significant effect size. The circle from the inside to the outside in **(B)** represents the classification level from phyla to genera. The diameters of the circles represent the relative abundance. Yellow indicates no significant difference; red, blue, and green dots represent significant differences in the pedigree for group C+P, group P, and group H (respectively).

To further understand the differences in microbial communities in the different groups, we undertook linear discriminant analysis (LDA) coupled with effect size (LEfSe) analyses with an LDA value of 3.0. Forty-six taxa were found to be significantly different in patients with COPD and periodontitis, in patients with periodontitis alone, and in HCs. Actinobacteria had the highest LDA value in the periodontitis + COPD group. Peptostreptococcaceae had the highest value in the periodontitis group and Fusobacterium had the highest value in HCs (Figure 5A). Significant changes in the Actinobacteria phylum, Negativicutes class, were noted at all taxonomic levels down to genera (Figure 5B).

Figure 5A shows the bacterial clades that were differentially abundant among the periodontitis (blue), periodontitis + COPD (red), and HC (green) groups. Clades in this graph were significant (*P* < 0.05) and had an LDA score > ±3, which was considered a significant effect size. The circle from inside to outside in Figure 5B represents the classification levels from phyla to genera. The diameter of the circles represents the relative abundance. Yellow denotes no significant difference; red, blue, and green dots represent significant differences of bacterial abundance and diversity in the periodontitis + COPD group, periodontitis-alone group, and HCs, respectively.

### General Trends of Significantly Different Bacterial Genera in the Microbiome Appeared in Patients of all Groups (Core Microbiome) Between Patients With Periodontitis With and Without COPD and HCs

**Figure 6:**
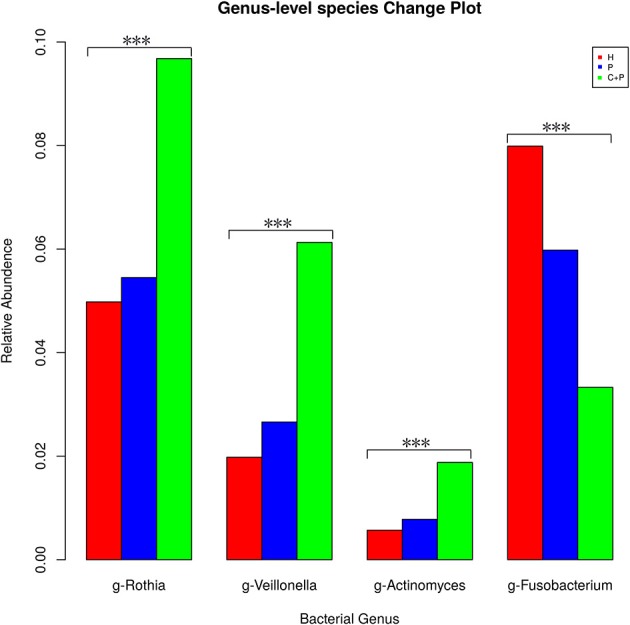
Mean relative abundance of the significantly changed core microbiome in saliva among healthy controls (H), patients with periodontitis (P), and patients with both periodontitis and COPD (C+P). *** Indicates significant differences assessed using the Kruskal–Wallis test (*p* < 0.05).

Among the 23 OTUs that appeared in every single sample (from all groups), we found four bacterial genera that were significant (Kruskal–Wallis test, *p* < 0.05) and that followed a general trend among the groups. *Rothia* and *Actinomyces* were the most unstable genera among the phylum Actinobacteria, and the same was true of *Veillonella* in the phylum Firmicutes. All three were more abundant in the periodontitis-alone group compared with that in HCs and were even more abundant in the periodontitis + COPD group. *Fusobacterium* displayed the opposite trend, being detected less frequently in patients with periodontitis and much less frequently in patients with periodontitis and COPD (Figure 6).

### Statistical Analyses of the Relative Abundance of *Rothia, Vellonella*, and *Actinomyces* in Clinical Subgroups of Sex, Smoking, PLI, and BI Between Disease Groups and HCs

**Figure 7:**
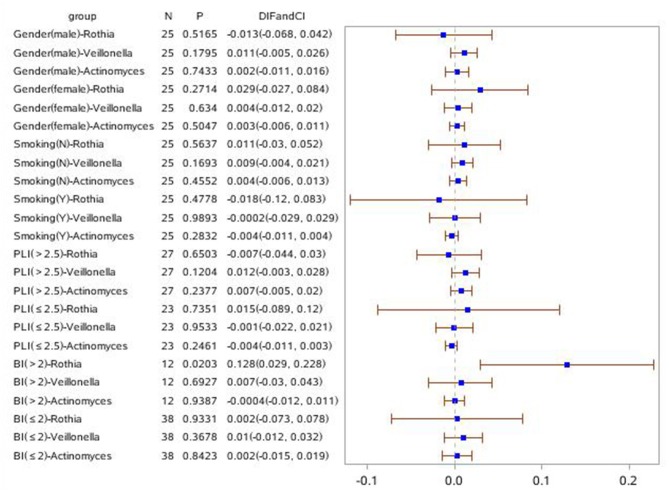
Forest Plot comparing the relative abundance of Rothia, Vellonella and Actinomycess bacterial in subgroups of gender, smoking, PLI and BI index among periodontitis and healthy control population.

**Figure 8:**
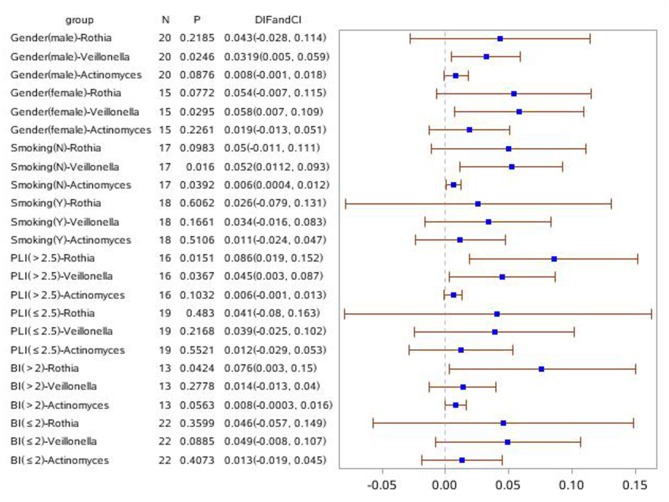
Forest Plot comparing the relative abundance of Rothia, Vellonella and Actinomycess bacterial in subgroups of gender, smoking, PLI and BI index among periodontitis with COPD and healthy control population.

In order to investigate the relationship of clinical symptoms with the three bacteria mentioned above, two forest plots have been drawn to compare periodontitis patients with and without COPD to healthy controls in clinical subgroups (Figures 7, 8). Figure 7 shows that *Rothia* was significantly different between the periodontitis group and HCs in the subgroup of BI > 2 (mean value). In the plot for periodontitis + COPD vs. HCs, the same trend for *Rothia* was observed. In addition, *Rothia* and *Vellonella* were significantly different in the subgroup of PLI >2.5 (mean value) and *Vellonella* was significantly different in subgroups of female, male and non-smoking populations.

## Discussion

We used 16s rRNA gene sequencing to analyze salivary microbial communities in patients with COPD and periodontitis, patients with periodontitis alone, and in HCs. Among all the bacteria that appeared only in one group, Lachnospiraceae were observed most frequently in samples from the COPD + periodontitis group, suggesting that they newly colonize only people with COPD and periodontitis. This genus may be a “signature” for patients with both diseases. Bacterial phylogenetic diversities were significantly different in patients with COPD, suggesting that patients with COPD have a different saliva bacterial ecology that may be caused by changes in the saliva microenvironment. *Veillonella, Rothia, Actinomyces*, and *Fusobacterium* were identified as the four core bacterial genera with significant differences among patients with COPD and periodontitis, patients with periodontitis alone, and HCs. Levels of *Veillonella, Rothia*, and *Actinomyces* tended to increase in the periodontitis group and increased even further in the COPD + periodontitis group. *Fusobacterium* levels tended to decrease in the periodontitis group and decreased even more in the COPD + periodontitis group. This trend suggested that, on the one hand, COPD aggravated periodontitis symptoms but, on the other hand, suggested the possibility of improving COPD symptoms by treating periodontitis.

The microbiome in humans can be classified into a “core” microbiome shared among all individuals comprising the predominant species in health at different anatomic sites (Turnbaugh et al., 2007; Zaura et al., 2009; Sonnenburg and Fischbach, 2011). There must be a reason why the relatively stable variability and diversity remains balanced among healthy populations. In the host, disturbance of this balance by changing the abundance and diversity of species might alter the tolerance to toxins produced directly by the microbiota and increase the systemic inflammatory response (e.g., circulating cytokines), which may be beyond the tolerance of blood capillaries over terminal alveoli (Wade, 2013). Thus, achieving a new balance among the changed salivary bacteria or a return to the original balance would appear to be rational ways of solving this problem.

The four core microbiome members we identified as having a significant difference in abundance compared with that in HCs, have previously been reported in saliva. *Rothia mucilaginosa* is a Gram-positive coccus of the family Micrococcaceae. *R. mucilaginosa* is considered as part of the healthy microflora of the human mouth and upper respiratory tract. Despite its low virulence, it is recognized as an opportunistic pathogen that mostly affects immunocompromised hosts (Maraki and Papadakis, 2015). In a study by Lim et al. *R. mucilaginosa* was an opportunist pathogen in 83% of cystic-fibrosis patients, and was almost as prevalent as *Pseudomonas aeruginosa* (89%) (Lim et al., 2013). Thus, in immunocompromised and immunocompetent hosts, *R. mucilaginosa* could be used to diagnose pneumonia. A strain isolated from the tongue plaque of a healthy human adult has been reported to contain the complete genome sequence of *R. mucilaginosa* NUM-Rm6536 (Nambu et al., 2015). This strain (which is amenable to genetic manipulation by transformation) can be used to provide more detailed information of this species. Ng et al. found that *Rothia dentocariosa* was 2.6% (range, 1.0–4.6%) of the total bacteria of 8.0 × 10^7^ CFU/ml on BHI in oral cavities (Ng et al., 2013).

Traditionally, the anaerobic bacteria *Veillonella* have been considered to be non-pathogenic flora. *V. atypica, V. dispar, V. denticariosi, V*. *rogosae, V. parvula*, and *V. tobetsuensis* are early colonizers in oral biofilm formation (Mashima and Nakazawa, 2014). *V. parvula* with high genetic variability within strains of the same species is considered to be the predominant subgingival *Veillonella* species. Subgingival *V. parvula* can translocate to the lungs (Leuckfeld et al., 2010). Nour et al. reported that a 63-years-old-male had an unusual presentation of multiple lung abscesses. The probable diagnosis was a periodontal disease associated-septic pulmonary embolism (PD-SPE), and *Veillonella* species were implicated (Abel Nour et al., 2013).

In the oral cavity, *Actinomyces* species are regular resident microbiota. *A. dentalis* is found at higher percentages in periodontitis patients, and *A. massiliensis* and *A. gerencseriae* have been found significantly more often supragingivally than subgingivally in people with periodontitis (Vielkind et al., 2015). Peptidoglycans from *A. naeslundii* can stimulate osteoclastogenesis in alveolar bone resorption and induce production of inflammatory cytokines (Sato et al., 2012). In a study by Endo et al. a case of PD-SPE was identified by anaerobic culture of bronchoalveolar lavage fluid and the culprit was suspected to be *Actinomyces* species. The symptoms could not be treated with antibacterial therapy alone, and additional dental treatment was indicated (Endo et al., 2015).

The same four bacterial species have also been found to be members of the core microbiome in sputum. The microbiota in patients with stable COPD was analyzed using pyrosequencing by Aguirre and coworkers. They found that in sputum, the prevalent genera were *Streptococcus, Neisseria, Actinomyces, Rothia, Haemophilus, Gemella, Granulicatella, Fusobacterium, Porphyromonas, Veillonella*, and *Prevotella* (Aguirre et al., 2015).

Our results showed that saliva levels of *Rothia, Veillonella*, and *Actinomyces* increased significantly during periodontitis, and increased even more in patients with periodontitis and COPD. In addition, the saliva level of *Fusobacterium* decreased significantly during periodontitis, and decreased even more in patients with periodontitis and COPD. Thus, the disturbed balance of the core microbiome in saliva may be a signal of lung disease. It has been reported that *Prevotella, Acinetobacter, Rothia, Neisseria, Leptotrichia, Lactobacillus, Veillonella, Streptococcus*, and *Actinomyces* are the most common genera in sputum during exacerbations of severe COPD (Su et al., 2015). The most prevalent phyla in the sputum of patients with stable COPD have been reported to be Proteobacteria and Firmicutes, followed by Actinobacteria (Garcia-Nunez et al., 2014). Thus, monitoring the changes in the abundance of these four core genera may aid early detection of the exacerbations of severe COPD.

Furthermore, two forest plots were drawn to clarify the relationship between bacterial and clinical symptoms of periodontitis with and without COPD. *Rothia* was meaningful in a BI > 2 (mean value) subgroup compared with periodontitis with and without COPD and HC groups. In the PLI > 2.5 (mean value) population, the relative abundance of *Rothia* and *Vellonella* was increased significantly in the periodontitis + COPD group compared with that in HCs. Thus, for a patient with gingival bleeding, the relative abundance of *Rothia* is meaningful; for a patient with obvious tooth plague, the relative abundance of *Rothia* and *Vellonella* may indicate the severity of inflammation of periodontitis and COPD. Our further research can be designed based on the relationship between bacteria and these clinical symptoms to predict the severity of periodontitis with and without COPD.

Recent research has suggested that, as well as COPD, periodontitis is related to several chronic systemic diseases: rheumatoid arthritis (Scher et al., 2014), diabetes mellitus (Wang et al., 2014; Chrysanthakopoulos and Chrysanthakopoulos, 2016), and cardiovascular diseases. Patients with periodontitis have been observed to be at an increased risk of cardiovascular disease (Dietrich et al., 2013; Widen et al., 2016), hypertension (Chrysanthakopoulos and Chrysanthakopoulos, 2016) and stroke (Sfyroeras et al., 2012; Chrysanthakopoulos and Chrysanthakopoulos, 2016). The change in the bacterial ecology of saliva is aggravated in patients with COPD. Therefore, restoring the original balance of the microbiome may be a way of controlling the severity of, or reducing the frequency of COPD exacerbations. Periodontal treatment of patients with common comorbidities could reduce costs and hospital admissions for diabetes mellitus and cardiovascular disease (Jeffcoat et al., 2014). We investigated whether treating periodontitis could cure COPD, and found that periodontal treatment in patients with periodontitis and COPD might reduce the frequency of COPD exacerbations and a decline in lung function (Zhou et al., 2014). Identification of four core microbiome members in the present study could lead to improved treatments for periodontitis and COPD.

## Conclusions

We identified significant differences among the salivary microbiota in patients with COPD and periodontitis, patients with periodontitis alone, and HCs. These findings provide clues for further functional studies to identify the mechanisms underlying pathogenic chronic inflammation in the mouth as well as the systemic inflammatory processes in other organs.

## Data Availability Statement

The datasets GENERATED for this study can be found in NCBI Sequence Read Archive (SRA), accession PRJNA578492.

## Ethics Statement

The studies involving human participants were reviewed and approved by The human research ethical board from Beijing Chao-Yang hospital approved the study (2018–KE292). The patients/participants provided their written informed consent to participate in this study.

## Author Contributions

SW, ZW, and ML designed the study. ML and XL interpreted the data and wrote the paper. ML, JW, CC, and TZ gathered clinical data. SW, ZW, ML, XL, XH, and YS conducted data analysis.

### Conflict of Interest

The authors declare that the research was conducted in the absence of any commercial or financial relationships that could be construed as a potential conflict of interest.
